# Immunoparesis in multiple myeloma: a current overview

**DOI:** 10.3389/fonc.2025.1628144

**Published:** 2025-09-23

**Authors:** Hongfeng Wang, Jianbin Chen

**Affiliations:** Department of Hematology, The First Affiliated Hospital of Chongqing Medical University, Chongqing, China

**Keywords:** multiple myeloma, immunoparesis, bone marrow microenvironment, prognosis, infections, immune reconstitution

## Abstract

Multiple myeloma (MM) is a common hematologic malignancy characterized by clonal plasma cell proliferation. Despite significant therapeutic advancements with proteasome inhibitors, immunomodulatory drugs, and anti-B-cell maturation antigen (BCMA) therapies, the disease remains largely incurable. Immunoparesis, a severe state of immune dysfunction, exhibits high prevalence in MM patients and profoundly impacts prognosis. This review summarizes the pathogenic mechanisms and clinical characteristics of immunoparalysis, with a focus on its impact on prognosis and early-onset infections, the effects of contemporary drug therapies on immunoparalysis, and immune reconstitution.

## 1 Introduction

Multiple myeloma (MM) is the second most common hematologic malignancy, accounting for 10% of hematologic cancers (1, 2), with a median diagnosis age of 65 years (3). This disease predominantly affects older adults and is characterized by malignant plasma cells that typically secrete monoclonal immunoglobulin, leading to end-organ damage, including anemia, renal impairment, osteolytic lesions, and hypercalcemia. Autologous stem cell transplantation (ASCT) remains the frontline treatment for MM. Meanwhile, the advent of novel agents and advancements in immunotherapy, particularly T-cell redirecting immunotherapies such as bispecific antibodies and chimeric antigen receptor (CAR) T-cell therapies, have significantly improved survival outcomes (4). Nevertheless, MM remains largely incurable, with infections still contributing significantly to early mortality (5).

Immunoparesis, the suppression of one or more uninvolved immunoglobulins (i.e., polyclonal immunoglobulins) in MM patients, such as the reduction of IgA and/or IgM levels in IgG MM patient, is a hallmark of MM and its precursor states, monoclonal gammopathy of undetermined significance (MGUS) and smoldering multiple myeloma (SMM) (6, 7).It occurs in 80%–95% of MM patients (8, 9) and correlates with poorer treatment outcomes, including reduced response depth and survival. Notably, immunoparesis is not limited to a decrease in immunoglobulin levels but can also involve multiple immunoglobulin isotypes. Reconstitution of polyclonal immunoglobulins serves as a dynamic marker for assessing treatment efficacy and long-term outcomes (10, 11). Furthermore, immunoparesis significantly increases the risk of infections in MM patients, complicating their clinical management. This review discusses the pathogenesis of immunoparesis, its clinical features, its associations with cytogenetic abnormalities, and its prognostic implications. Additionally, we examine the impact of immunoparesis on infection risk, its recovery and reconstruction, and the effects of modern therapeutic regimens on immunoparesis. Lastly, we propose future research directions to enhance the understanding of immunoparesis in the context of MM.

Relevant studies were identified by searching PubMed and Web of Science (2000–2024) using keywords such as “immunoparesis”, “multiple myeloma”, “immune reconstitution”, “immunoglobulin suppression” and “immune recovery”.

## 2 Pathogenesis and cytogenetic interplay of immunoparesis in multiple myeloma

### 2.1 Mechanisms of immunoparesis

The pathogenesis of MM is complex, involving genetic mutations (12, 13), chromosomal translocations (14–16), aberrant signaling pathways (17, 18), and a supportive bone marrow microenvironment (13, 19, 20). Key oncogenic events include IgH translocations, such as t(11;14) and t(4;14), as well as chromosomal abnormalities like 1q gain and 13q deletion. These alterations promote cell cycle dysregulation and clonal expansion. In addition, disease progression is also influenced by interactions between myeloma cells and the bone marrow microenvironment. These interactions stimulate the secretion of cytokines and inflammatory mediators, including interleukin-6 (IL-6), insulin-like growth factor-1 (IGF-1), B-cell activating factor (BAFF), a proliferation-inducing ligand (APRIL), tumor necrosis factor-α (TNF-α), and vascular endothelial growth factor (VEGF). These soluble factors activate multiple signaling pathways, including NF-κB, JAK/STAT, MAPK, and PI3K/Akt/mTOR, which promote myeloma cell proliferation, survival, migration, and resistance to therapy (19, 21, 22).

**Figure 1:**
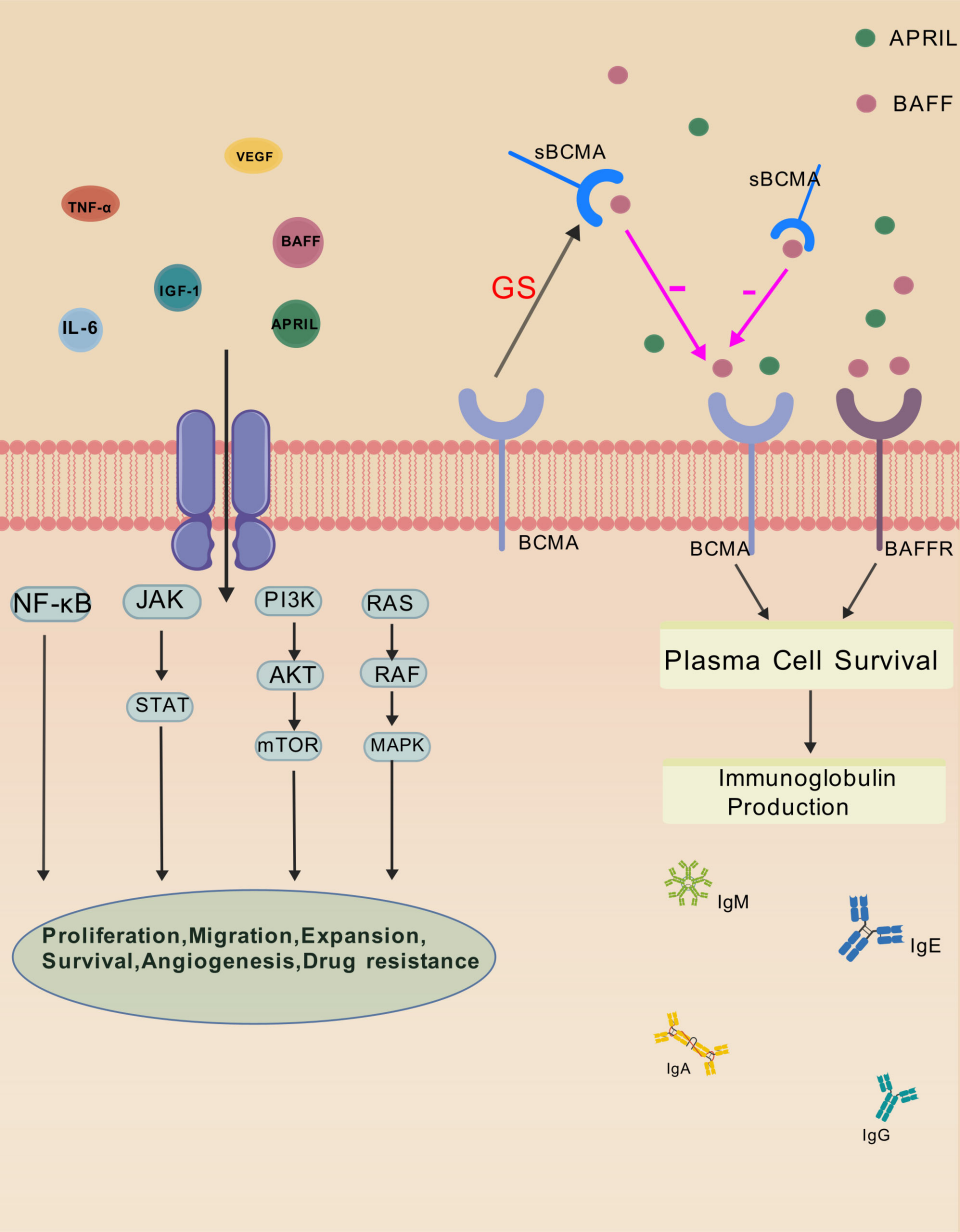
Mechanisms of Immunoparesis in multiple myeloma. Cytokines produced through interactions between myeloma cells and the bone marrow microenvironment, such as IL-6, IGF-1, BAFF, APRIL, TNF-α, and VEGF, activate signaling pathways including NF-κB, JAK/STAT, RAS/MAPK, and PI3K/Akt/mTOR. These pathways promote myeloma cell proliferation, survival, migration, angiogenesis, and drug resistance. BCMA is highly expressed on plasma cells and binds BAFF and APRIL to support plasma cell survival and polyclonal immunoglobulin production. γ-secretase(GS) cleaves membrane-bound BCMA to generate soluble BCMA (sBCMA), which competes with membrane BCMA for binding to BAFF. This disrupts BCMA signaling, impairs plasma cell function, reduces immunoglobulin synthesis, and contributes to immunoparesis in multiple myeloma.

Recent studies suggest that immunoparesis in MM may be closely related to interactions between anti-B-cell maturation antigen (BCMA) and its ligands, BAFF and APRIL (23, 24).BCMA, a transmembrane glycoprotein belonging to the tumor necrosis factor (TNF) receptor superfamily, is highly expressed on both normal and malignant plasma cells and serves as a hallmark surface antigen (25). Upon binding with BAFF or APRIL, BCMA promotes plasma cell survival, proliferation, and polyclonal immunoglobulin secretion (26–28). However, membrane-bound BCMA can be cleaved by γ-secretase(GS), a multi-subunit protease complex, to generate soluble BCMA (sBCMA), which is released into the peripheral blood (27, 29). sBCMA competitively bind to BAFF and APRIL, thereby blocking their interaction with membrane-bound BCMA. This interference disrupts the plasma cell maturation and function, leading to impaired immunoglobulin synthesis and secretion, and ultimately contributing to immune dysfunction and the development of immunoparesis. The above mechanisms are shown in **Figure 1** (30).

### 2.2 Immunoparesis and cytogenetic abnormalities

Cytogenetic abnormalities are among the most common predictors of poor prognosis in MM (31–34). High-risk abnormalities, such as t(4;14), t(14;16), t(14;20), ≥3 copies of the 1q21 chromosomal band (1q21+), deletions of the 1p chromosomal arm (del(1p)), and deletions of the 17p13 chromosomal band (del(17p)) (35), are strongly associated with disease progression. Evidence suggests that in newly diagnosed MM patients, primary cytogenetic abnormalities intensify the severity of immunoparesis. Specifically, patients with high-risk alterations, including t(4;14), t(14;16), or t(14;20), had a significantly higher proportion of polyclonal immunoglobulins suppressed below the normal range. Mechanistically, these cytogenetic abnormalities alter the bone marrow microenvironment, remodel the plasma cell niche, and suppress normal plasma cell function, ultimately reduce immunoglobulin production (36).But to date, no more comparable studies have been reported in this field.

## 3 Incidence and clinical features of immunoparesis

Immunoparesis is more prevalent in MM patients aged >65 years (37), with a reported incidence of 85%–90% in newly diagnosed cases (3, 11). The incidence further increases to 90%–95% in relapsed/refractory MM (RRMM) (7), likely due to enhanced immune dysfunction caused by tumor resistance and immune escape. The prevalence of immunoparesis correlates with disease progression; early-stage MM exhibits a lower incidence (50%–60%), whereas advanced-stage MM demonstrates rates exceeding 90%. Specifically, among active MM cases, immunoparesis prevalence rises from 63% in Durie-Salmon (D-S) stage I to 90% in stage III (38). A Greek study reported immunoparesis rates of 77%, 88%, and 94% in International Staging System (ISS) stages 1, 2, and 3, respectively (9).

Moreover, immunoparesis is significantly associated with laboratory abnormalities, including decreased hemoglobin levels, reduced platelet counts, lower M-protein levels, and increased infiltration of bone marrow plasma cells(BMPC) (9, 39). While it occurs across all M-protein subtypes, studies offer conflicting conclusions regarding which subtype is most closely associated. A small retrospective study (n = 49) found that patients with IgG-type MM had significantly lower levels of polyclonal immunoglobulins compared to those with IgA or IgM types, suggesting stronger isotype-specific immunosuppression in the IgG subtype (40). This study utilized serum electrophoresis, which is limited in differentiating monoclonal from polyclonal immunoglobulins. In contrast, a larger prospective cohort study (n = 1,755) using nephelometry reported a higher incidence of immunoparesis in patients with IgA-type MM than those with IgG-type (9). This association remained significant after multivariate regression analysis. We speculate that differences in cohort size and testing methodology account for the discrepancies between the two studies.

Emerging evidence suggests that immunoparesis is not only a clinical feature of MGUS and SMM but may also drive disease progression. In MGUS patients, suppression of two or more uninvolved immunoglobulins significantly increased the risk of progression to MM (OR = 19.1), with prognostic value comparable to the IgA subtype, elevated M-protein, and abnormal free light chain ratios (41). Moreover, the combination of immunoparesis and an abnormal bone marrow plasma cell phenotype helps identify high-risk MGUS patients, with a 5-year progression risk of up to 46% (42). In SMM, deeper immunoglobulin suppression has similarly been linked to faster progression (43). Specifically, patients with suppression of two immunoglobulin types experienced a median time to progression of only 32 months, compared to 159 months in those without suppression. Additionally, some researchers incorporated immunoparesis into their high-risk SMM evaluation model, reflecting its association with increased tumor burden (44).

## 4 Prognostic and infection-related consequences of immunoparalysis in multiple myeloma

### 4.1 Impact on prognosis

Despite significant advancements in therapeutic strategies for MM, the disease remains largely incurable, with a 10-year survival rate of merely 17% (45, 46). This underscores the pressing need for a more nuanced understanding of prognostic factors and the establishment of reliable risk stratification systems to better identify high-risk patients, guide treatment intensification, and improve prognostic accuracy. To date, several prognostic systems, including the International Staging System (ISS) (47) and its revised version, the Revised International Staging System (R-ISS) (48), have been established. The R-ISS integrates key factors such as β2-microglobulin, lactate dehydrogenase (LDH), and high-risk cytogenetic abnormalities. More recently, the introduction of sensitive markers like minimal residual disease (MRD) detection has improved clinical practice (45, 49). Research consistently shows that immunoparesis is significantly associated with inferior survival in MM patients, with greater severity correlating with worse outcomes (9, 50).These findings suggest that immunoparesis may serve as a valuable predictive biomarker for MM prognosis. **Table 1** offers a comprehensive summary of the effects of immunoparesis on the prognosis of MM patients.

**Table 1:** Studies investigating the prognostic impact of immunoparalysis in patients with multiple myeloma.

Study (author, year)	Cohort (n)	OS		PFS		Reference
		With immunoparesis	No immunoparesis	With immunoparesis	No immunoparesis	
Kastritis,et al., 2014	1755	41.5months	55months	25months	60months	(9)
Chakraborty,et al., 2020	258	3‐year OS 40%-42%	3‐year OS 60%	2‐year PFS 20%-25%	2‐year PFS 36%	(7)
Gao,et al., 2019	108	estimated OS of not reach	61months	32months	55months	(51)
Geng,et al., 2021	287	45.6months	30.9months	27.6months	25.6months	(50)
González-Calle,et al., 2017	169	7.3years	11.3years	27.9 months	60.4months	(11)

#### 4.1.1 For newly diagnosed multiple myeloma patients

At diagnosis, patients with immunoparesis typically experience worse overall survival (OS) and progression-free survival (PFS) compared to those without it (38). In a cohort of 1,755 newly diagnosed multiple myeloma (NDMM) patients, reported preserved uninvolved immunoglobulins correlated with longer median OS compared to those with suppressed levels (55 vs. 41.5 months, *P < 0.001*), and immunoparesis remained an independent prognostic factor in multivariate analysis (HR = 0.781, *P = 0.039*) (9). Geng et al. classified immunoparesis as deep (any uninvolved immunoglobulin <50% of the lower normal limit) or partial (≥2 suppressed isotypes), both of which were linked to significantly worse OS and PFS even after propensity score matching (50). However, the study was limited by its single-center, retrospective design and relatively small sample size. Although propensity matching helped control confounding factors, selection bias could not be fully excluded. Additionally, a larger retrospective study analyzed 2,558 NDMM patients by stratifying them into three groups based on the degree of polyclonal IgM suppression. Median PFS decreased with increasing IgM suppression—1.97, 1.79, and 1.71 years across the least, intermediate, and most suppressed groups, respectively (*P < 0.001*). No similar trend was observed for IgG or IgA (52). These findings suggest that IgM may serve as a more sensitive prognostic marker, though this could also relate to its lower baseline concentration and greater susceptibility to decline.

#### 4.1.2 For relapsed multiple myeloma

In relapsed MM, immunoparesis remains associated with adverse outcomes, particularly at first relapse where it often coincides with higher tumor burden and shorter remission durations (7, 53). A study of 258 patients with first relapse stratified immunoparesis both qualitatively and quantitatively (7). Qualitatively, patients were categorized into three groups: no immunoparesis (no suppression of uninvolved immunoglobulins), partial immunoparesis (suppression of at least one but not all), and complete immunoparesis (suppression of all uninvolved immunoglobulins). Although OS did not differ significantly among groups, 2-year PFS declined progressively (36%, 25%, and 20%), suggesting a strong link between the extent of immunoparesis and disease progression. Quantitative analysis used the average relative difference (ARD) between polyclonal immunoglobulin levels and the lower limit of normal, categorizing patients into no immunoparesis (ARD ≥ 0%), mild immunoparesis (ARD 0%−50%), and deep immunoparesis (ARD ≤−50%). Patients with deep immunoparesis had significantly worse OS and PFS compared to those with mild or no suppression. Interestingly, only IgM suppression showed a statistically significant association with prognosis, consistent with findings in newly diagnosed cohorts. This supports the hypothesis that IgM may be a more sensitive indicator of impaired immune surveillance.

In summary, immunoparesis is associated with poor outcomes in both newly diagnosed and relapsed MM, with IgM suppression showing consistent prognostic relevance across studies. However, most supporting data are retrospective and affected by heterogeneity in patient populations, definitions, treatment regimens, transplant status, and maintenance therapy, which may contribute to inconsistent results. Although multivariate analyses (e.g., HR = 0.781, *P = 0.039*) support its independent prognostic value, immunoglobulin suppression as a standalone marker has limitations and may be influenced by disease burden and treatment response. Future research should aim to standardize grading criteria and assess its additive value within multifactorial models.

### 4.2 Impact on infections

Infections are frequent and serious complications in MM, accounting for a substantial proportion of early morbidity and mortality (54–57). Approximately 45% of deaths within the first six months following diagnosis are attributed to infections (39, 58), with the highest risk occurring during the initial post-diagnosis period and treatment for relapsed or refractory disease (54, 59, 60). Modern therapies, including proteasome inhibitors, immunomodulatory drugs, and monoclonal antibodies, may further weaken host immune defenses, thereby increasing infection risk. Consequently, infection prevention and management are critical in MM treatment.

Immunoparesis, which impairs humoral immunity, increases susceptibility to infections (61). A recent retrospective study involving 430 newly diagnosed patients reported that 59.5% experienced ≥Grade 3 infections within the first three months. Respiratory infections predominated, and bacterial infections constituted the majority of identified cases, findings consistent with observations from comparable studies (62). Patients with immunoparesis exhibited significantly higher early infection rates compared to those without immunoglobulin suppression. Based on these findings, a predictive model incorporating immunoparesis was developed to assess early infection risk in MM, suggesting that immunoparesis may negatively impact prognosis by increasing susceptibility to infections. In contrast, other studies argue that the adverse prognostic impact of immunoparesis cannot be primarily explained by elevated infection rates (38). Thus, the role of immunoparesis in mediating poor outcomes through infection remains inconclusive and warrants further investigation.

## 5 Recovery and reconstitution of immunoparesis

Immunoparesis is recognized as a poor prognostic indicator in MM, prompting researchers to explore whether reversing this condition during treatment can lead to clinical benefits. Several studies have examined the restoration of polyclonal immunoglobulins in various therapeutic contexts (11, 37, 51). In particular, patients undergoing ASCT often show a gradual recovery of immunoglobulin levels within the first year (51). This increase may reflect the reconstitution of the immune system and could be linked to improved long-term outcomes.

Analysis of a 295-patient ASCT cohort revealed that individuals achieving immunoglobulin recovery within one year had significantly improved PFS (60.4 vs. 27.9 months) and OS (11.3 vs. 7.3 years) compared to those without recovery (11). Earlier recovery was linked to better outcomes, with median PFS of 69.3, 52.9, and 27.9 months for patients recovering within 6 months, between 6–12 months, and those who did not recover within one year, respectively (*P < 0.001*). Additionally, a higher proportion of normal plasma cells (nPCs) in bone marrow at day 100 post- ASCT was associated with immunoglobulin recovery, suggesting that early nPC reconstitution may serve as a predictive marker for subsequent immune restoration, consistent with observations reported in other studies (63).

Recovery patterns have also been noted in patients undergoing allogeneic stem cell transplantation (alloSCT) and found that a majority experienced gradual recovery of polyclonal immunoglobulins within one year, particularly those with non-relapsing patients (64). These findings highlight the potential clinical relevance of immune reconstitution after alloSCT. Nonetheless, due to the small sample size and lack of multivariate analysis, the conclusions remain exploratory.

In newly diagnosed MM patients not undergoing transplantation, polyclonal immunoglobulin recovery during therapy has similarly been linked to improved survival (65). Even after adjusting for known prognostic variables such as ISS stage, LDH, response depth, and treatment regimen, this association remained statistically significant, underscoring the independent prognostic value of immune reconstitution.

Overall, immunoglobulin recovery correlates with improved survival in MM patients, but the variability across studies, patient populations, and treatment regimens calls for further research. Future studies should standardize definitions of immunoglobulin recovery and integrate it with other prognostic factors such as MRD and immune phenotyping to clarify its role in multifactorial prognostic models.

## 6 Impact of modern therapeutic regimens on immunoparesis

Current frontline pharmacologic treatment strategies for MM primarily rely on combination regimens that include proteasome inhibitors (e.g., bortezomib, V), immunomodulatory agents (e.g., lenalidomide, R), corticosteroids, and monoclonal antibodies (e.g., daratumumab, D) (66, 67). These therapies have significantly improved response depth and patient survival, and their effects on immune function are gaining increasing attention. Recent studies have demonstrated that induction regimens based on V and R, such as RD, VD, and VRD, not only effectively reduce tumor burden but also promote the recovery of uninvolved immunoglobulins, suggesting a potential role in alleviating immunoparesis (24). This effect may be attributed to the ability of R and V to inhibit the BAFF/APRIL/BCMA signaling axis (68).

In parallel, therapeutic strategies targeting soluble B-cell maturation antigen (sBCMA) present novel opportunities for mitigating immune dysfunction. Preclinical animal studies have shown that small-molecule γ-secretase inhibitors (GSIs) can block BCMA shedding, enhance its membrane-bound expression, and reduce circulating sBCMA levels, thereby improving CAR-T cell recognition and cytotoxicity against MM cells (29). However, whether GSIs can independently alleviate immunoparesis by reducing sBCMA production or enhance long-term prognosis, remains to be determined. Future studies should explore the immunomodulatory effects of GSIs outside of CAR-T therapeutic contexts, particularly their ability to restore uninvolved immunoglobulin production and reverse immunoparesis.

## 7 Summary and perspectives

Immunoparesis, defined by suppression of uninvolved polyclonal immunoglobulins, is common in MM. It is strongly associated with inferior prognosis and increased infection risk. Evidence shows that early recovery of uninvolved immunoglobulins, particularly within one year post-ASCT, correlates with improved PFS and OS. Therefore, dynamic monitoring of immunoglobulin levels during treatment may serve as an important tool for assessing immune reconstitution and long-term treatment response.

Future efforts should aim to incorporate immunoparesis into established prognostic models such as ISS, R-ISS, MRD status, and immune phenotyping. This could help develop composite scoring systems that combine clinical and immune-related factors. We recommend establishing a standardized stratification framework for immunoparesis based on both qualitative and quantitative criteria to improve cross-study comparability and clarify its prognostic relevance. Immunoparesis may serve as a surrogate marker for immune competence. Future studies could use it to stratify patients and predict immune responses in vaccine trials, infection risk assessment, and CAR-T eligibility. Future prospective trials should consider using immunoglobulin recovery as a clinical endpoint to assess the immune effects of therapies such as IMiDs, monoclonal antibodies, or cellular therapies. Clinical studies could also integrate biomarkers based on immunoglobulin recovery into treatment decision-making and further investigate immunoparesis as a potential therapeutic target.
